# Echocardiography parameters used in identifying right ventricle dysfunction in preterm infants with early bronchopulmonary dysplasia: A scoping review

**DOI:** 10.3389/fped.2023.1114587

**Published:** 2023-03-20

**Authors:** Wisam Muhsen, Eirik Nestaas, Joanne Hosking, Jos M. Latour

**Affiliations:** ^1^ Faculty of Health, University of Plymouth, Plymouth, United Kingdom; ^2^ Neonatal Intensive Care Unit, University Hospitals Plymouth NHS Trust, Plymouth, United Kingdom; ^3^ Faculty of Medicine, University of Oslo, Oslo, Norway; ^4^ Clinic of Pediatrics and Adolesence, Akershus University Hospital, Nordbyhagen, Norway; ^5^ Medical Statistics, Faculty of Health, University of Plymouth, Plymouth, United Kingdom; ^6^ Faculty of Health Sciences, Curtin University, Perth, WA, Australia

**Keywords:** preterm infants, right ventricular function, bronchopulmonar dysplasia, echocardiogaphy, haemodynamic effects, scoping review

## Abstract

**Background:**

Bronchopulmonary Dysplasia (BPD) is a chronic condition that affects preterm infants and is associated with long-term complications. Haemodynamic effects of BPD can lead to right ventricular (RV) dysfunction.

**Objective:**

To synthesise and map the evidence of echo parameters used in identifying RV dysfunction in the first two weeks-after-birth (WAB) of preterm infants with early BPD.

**Information Sources:**

This scoping review included the databases: Medline, CINAHL, PubMed, EMBASE, Scopus, ProQuest, Web of Science, Cochrane Library, JBI Evidence-Based Practise and Gray Literature.

**Search Strategy:**

The search utilised Boolean operators and descriptors registered in Medical Subject Headings.

**Inclusion and exclusion criteria:**

Included were studies utilising echo parameters to examine RV function in preterm infants with early BPD in the first two WAB.

**Synthesis of results:**

The results are presented as a map of the extracted findings in a tabular format with a narrative summary.

**Results:**

Eight studies were included. Differences were observed in the number and timing of echo scans performed in the first two WAB and the variations in the echo parameters used to compare preterm infants with and without early BPD. Only echo scans performed at the end of the first WAB, demonstrated significant differences in the echo parameters measurements between preterm infants with and without BPD. Studies using RV Myocardial Performance Index (MPI) to identify RV-dysfunction associated with early BPD demonstrated similar findings. The Pulsed-Wave Doppler technique identified differences in RV-MPI between preterm infants with and without BPD, while Tissue-Doppler-Imaging did not demonstrate similar results. Speckle tracking can measure strain (S) and strain rate (SR) and diagnose RV-dysfunction. However, the findings of studies that utilised speckle tracking varied. Finally, two of the included studies added blood tests to their diagnostic model of early BPD, which was able to demonstrate significant differences in blood test results between BPD-affected and control preterm infants.

**Conclusion:**

BPD could adversely affect the myocardium function of the RV; these negative influences can be captured in the first two WAB. However, there are still knowledge gaps regarding the appropriate number, timing and the most suitable echo parameters to assess RV function.

## 1. Introduction

Bronchopulmonary Dysplasia (BPD) was first described in 1967 (1). It affects the immature lungs of preterm infants. Preterm infants born before 32 weeks of gestation are at a greater risk of developing BPD, especially those requiring respiratory support with higher oxygen requirements (2). The incidence rate of this disease remains high; a moderate and severe form of BPD affects almost a third of the preterm infants born before 32 weeks of gestation (3). The incidence rate of BPD is expected to continue to be elevated, especially when the viability threshold is reduced to 22 weeks of gestation, while more at-risk extreme preterm infants are expected to survive (4).

Bronchopulmonary Dysplasia negatively affects the normal growth and development of the immature lungs’ alveoli and vascular bed through complex processes, which can reduce gas exchange surfaces and subsequently result in a decline in pulmonary function (5). The vascular pathogenesis theory hypothesises that as the premature heart and the premature lungs' vascular bed are closely interlinked, a negative effect on one of them will also be reflected on the other. Abnormally affected lungs by early BPD will also impair angiogenesis of the vascular bed of the lungs and the formation of pulmonary vascular disease (PVD), which can adversely affect the function of the right ventricle (RV) of the heart.

The pulmonary vascular remodelling results in a rise in the vascular tone, altered reactivity, vasoconstriction, and increased pulmonary vascular resistance (6). These histologic changes can increase the RV afterload pressure. Subsequently, the chronic increase in RV afterload pressure and hypoxic episodes can result in RV dysfunction, hypertrophy and, in severe cases, failure (7). Furthermore, severe forms of BPD are associated with a higher incidence of pulmonary arterial hypertension (8), which results from an increase in pulmonary arterial pressure and is associated with significant co-morbidities and high mortality rates (9).

The literature showed that the pathological effects of early BPD and respiratory insufficiency could manifest as early as day 7 of postnatal life (10). Data from 1,735 infants born between 23 and 30 weeks of gestational age showed that the proportion of these preterm infants needing oxygen decreases from birth to day 7 of postnatal life, followed by a steep rise in the number of infants requiring oxygen in the same cohort at the start of the second week postnatal (10). Additionally, studies, albeit limited, such as by Czernik et al., 2012 (11), demonstrated that the negative effect of PVD associated with early BPD on the function of the RV is present as early as the first two weeks after birth.

To address the gap in knowledge and provide state-of-the-art current knowledge, the aim of this scoping review is to identify, synthesise and map the evidence of echo parameters used in identifying dysfunction of the RV in the first two weeks of postnatal life of preterm infants with early BPD. This review will examine the echo parameters in preterm infants born before 32 weeks of gestation since these infants are at higher risk of developing BPD. In addition, the review will include studies which performed echo scans in the first two weeks of postnatal life as it is the period when early BPD starts to manifest clinically. Specifically, the scoping review questions were: (i) What techniques are used to capture echo images in preterm infants? (ii) When are the echo scans performed in the first two weeks after birth? (iii) What echo parameters are used to assess the haemodynamic effects of early BPD on the function of the right ventricle? and (iv) How are the echo parameters measured and analysed?

## 2. Methods

The preliminary literature search on 01 April 2022 in Medline (OVID), Cochrane Library, PROSPERO and the JBI Evidence Synthesis Database revealed no scoping or systematic review available or currently being developed about this subject.

The proposed scoping review is conducted according to Joanna Briggs Institute (JBI) methodology (Peters M. D. J., 2020). Also, this scoping review used the reporting guideline “Preferred Reporting Items for Systematic Reviews and Meta-Analyses (PRISMA) extension for scoping reviews (PRISMA-ScR): checklist and explanation” (12).

### 2.1. Participants, concept and context

*Participants:* The scoping review included studies that examined the first two weeks of postnatal life of preterm infants born before 32 weeks of gestation.

*Concept:* The review included studies that examined the haemodynamic effects of PVD associated with early BPD on the RV function through the analyses of the echo parameters. The included studies should have at least one echo scan performed in the first two weeks after the birth of the participating preterm infants. In addition, eligible studies should have compared the data collected from echo parameters analyses in preterm infants with and without BPD.

*Context:* The review included studies that recruited preterm infants admitted to a neonatal intensive care unit.

### 2.2. Types of evidence and sources

This scoping review considered all study designs, including experimental and quasi-experimental studies. Randomised controlled trials, non-randomised controlled trials, before and after studies and interrupted time-series studies were assessed for eligibility. Any systematic reviews that meet the inclusion criteria were considered, but none were retrieved. The review also considered analytical observational studies, such as prospective and retrospective cohort studies, case-control studies and analytical cross-sectional studies. There were no limitations regarding the date of the publication.

Papers were excluded if they did not fit in the conceptual framework of this review. In addition, studies were excluded if they used different imaging modalities, such as Magnetic Resonance Imaging or Computed Tomography or were not available in full text. Studies that did not assess the RV function or echo scans performed after the first two weeks after birth were also excluded. Studies were excluded if they were unavailable in English or were animal studies.

### 2.3. Search terms

The selected text words and index terms, such as preterm infants, right ventricular function, bronchopulmonary dysplasia, and echocardiography, were utilised to formulate a full search strategy (Table 1).

**Table 1:** Search strategies. (Search completed on the 02nd of April 2022).

Database	Search strategy		Records retrieved
Medline (OVID)	((preterm infants or premature infants or preterm baby or premature baby) and bronchopulmonary dysplasia and (Right ventricular function or right ventricular dysfunction) and (echocardiography or echo or echocardiogram))		11
CINAHL Plus with Full Text (EBSCO*host*)	((preterm infants) OR (premature infants) OR (preterm baby) OR (premature baby)) AND (bronchopulmonary dysplasia) AND ((right ventricular function) OR (right ventricular dysfunction)) AND (echocardiography OR echo OR echocardiogram)		8
EMBASE	((preterm infants or premature infants or preterm baby or premature baby) and bronchopulmonary dysplasia and (Right ventricular function or right ventricular dysfunction) and (echocardiography or echo or echocardiogram))		15
Pubmed	(“preterm infants"[All Fields] OR “premature infants"[All Fields] OR “preterm baby"[All Fields] OR “premature baby"[All Fields]) AND “bronchopulmonary dysplasia"[All Fields] AND (“right ventricular function"[All Fields] OR “right ventricular dysfunction"[All Fields]) AND (“echocardiographies"[All Fields] OR “echocardiography"[MeSH Terms] OR “echocardiography"[All Fields] OR (“echo"[Journal] OR “echo"[All Fields]) OR (“echocardiography"[MeSH Terms] OR “echocardiography"[All Fields] OR “echocardiogram"[All Fields] OR “echocardiograms"[All Fields]))		14
Scopus	(Preterm AND infants OR premature AND infants OR preterm AND baby OR premature AND baby) AND (“bronchopulmonary dysplasia”) AND (right AND ventricular AND function OR right AND ventricular AND dysfunction) AND (echocardiography OR echo OR echocardiogram)		72
ProQuest	(preterm OR premature) AND (infant OR baby) AND (bronchopulmonary dysplasia) AND ((right ventricular function) OR (right ventricular dysfunction)) AND (echocardiography OR echo OR echocardiogram)		143
Web of Science	(((ALL = ((preterm infants) OR (premature infants) OR (preterm baby) OR (premature baby))) AND ALL = ((bronchopulmonary dysplasia))) AND ALL = ((right ventricular function) OR (right ventricular dysfunction))) AND ALL = (echocardiography OR echo OR echocardiogram)		51
Cochrane library	6.3 (preterm infants) OR (premature infants) OR (preterm baby) OR (premature baby) in Title Abstract Keyword AND (bronchopulmonary dysplasia) in Title Abstract Keyword AND (right ventricular function) OR (right ventricular dysfunction) in Title Abstract Keyword AND echocardiography OR echo OR echocardiogram in Title Abstract Keyword Words variations have been searched.		2
JBI EBP Database	((preterm infants or premature infants or preterm baby or premature baby) and bronchopulmonary dysplasia and (Right ventricular function or right ventricular dysfunction) and (echocardiography or echo or echocardiogram))		Zero
British Library—explore further	The following terms were searched with “Anywhere” option [Preterm infants OR premature infants OR preterm baby OR premature baby] AND [“right ventricular function” OR “right ventricular dysfunction” AND “echocardiography” OR “echo” OR “echocardiogram”] AND [“bronchopulmonary dysplasia”] Material type: all items No limitations on the date Search scope: explore further. Limitations: only human		35
EThOS E-thesis online service	"\(preterm infants\) OR \(premature infants\) OR \(preterm baby\) OR \(premature baby\)” AND “\(bronchopulmonary dysplasia\)” AND “\(right ventricular function\) OR \(right ventricular dysfunction\)” AND “echocardiography OR echo OR echocardiogram"		Zero
Google Scholar	with **all** of the words	Right ventricular	150
	with the **exact phrase**	bronchopulmonary dysplasia	
	with **at least one** of the words	function dysfunction preterm premature infants baby echocardiography echo echocardiogram	
	**without** the words	catheterization OR MRI OR CT	
	Where my words occur	Anywhere in the article	
Total number of articles in all the searched databases			501

### 2.4. Search strategy

A comprehensive search strategy was followed and consisted of three steps.

*First step:* An initial limited search of two online databases, Medline (OVID) and CINHAL Plus Full text (EBSCOhost), was conducted to allocate related articles. The examination of the relevant articles resulted in the identification of the text words included in the titles and the abstracts and the index terms related to these articles to guide the subsequent detailed search as per the advice of a research librarian.

*Second step:* The full search strategy was applied to the included databases with the identified keywords and index terms. The descriptors registered in the Medical Subject Headings (MeSH) were used wherever possible. The synonyms were combined with the Boolean operator “OR” while the groups of words were combined with the operator “AND” (Table 1). The following databases were searched: Medline (OVID), CINAHL Plus with Full Text (EBSCOhost), PubMed, EMBASE, Scopus, ProQuest, Web of Science, Cochrane Library, JBI Evidence Based Practise (EBP) and Gray Literature (British Library, google scholar & EThOS).

*Third step:* A reference list containing all the identified studies was formed. There were no scoping nor systematic reviews identified.

A full search was performed by two reviewers (WM and JML). Any unresolved dispute was discussed with a third reviewer (EN).

### 2.5. Studies selection

The studies were selected from the included databases. All the identified citations were uploaded into EndNote 20 (Clarivate Analytics, PA, USA), followed by removing duplicates. Then, studies were selected by two reviewers (WM and JML) in two stages; titles and abstracts review; and full-text review, against the inclusion criteria. A detailed assessment of the full text of the selected articles against the inclusion criteria was performed by two independent reviewers (WM and JML). Any disagreements were discussed and resolved by both reviewers.

### 2.6. Data extraction

Two independent reviewers (WM and JL) performed the data extraction process using the tool developed by the reviewers for this purpose. The data extraction tool contained details of the population, concept, context, methodology and relevant findings. No modifications to the data extraction tool were performed.

## 3. Results

**Figure 1:**
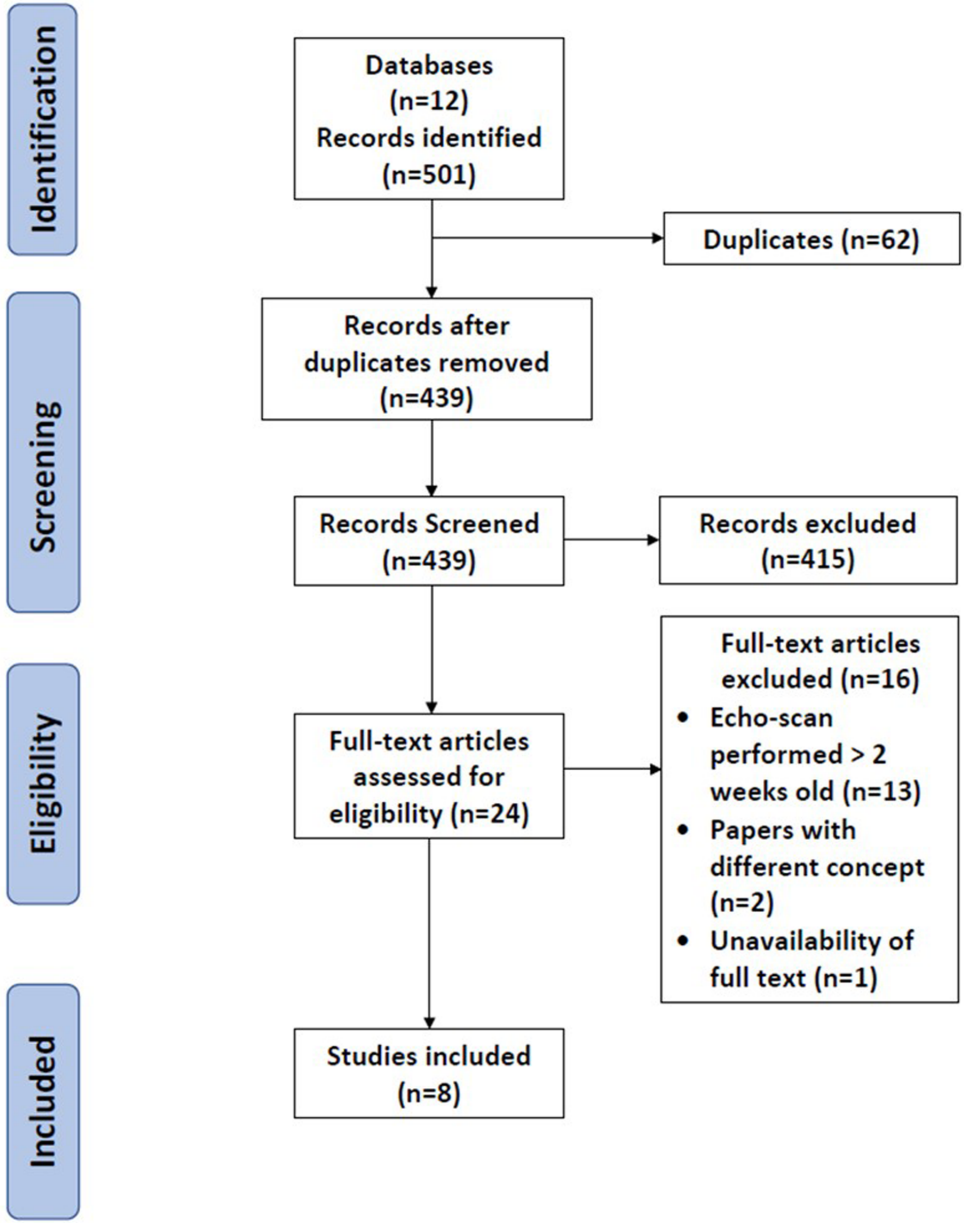
PRISMA flow diagram.

The final scoping review full report of the search results is presented in the PRISMA flow diagram (Figure 1). There were 501 records identified from 12 databases*.* Sixty-two duplicates were removed, leaving 439 records eligible for the first screening stage, i.e., titles and abstracts were screened. After the exclusion of 415 records, only 24 papers were eligible for the second stage of full-text screening. Sixteen articles were excluded (Figure 1), and eight studies were included in the final critical appraisal and analysis.

### 3.1. Data charting

Data were extracted from the full texts of the eight included studies and detailed into two tables (Tables 2, 3). Table one described the studies' methodologies, while table two included details about the echo scans' timing, echo parameters analyses and the key findings. The results are presented in narrative descriptions and mapping of the data.

**Table 2:** Selected studies methodologies.

Authors & year	Country of origin	Aims	Population & sample size	Settings & methodology
Bokiniec et al., 2017	Poland	Evaluation of right ventricular function in preterm infants with & without BPD	A total of 89 preterm infants (<32 weeks gestation) were included: – No-BPD *n* = 32 – Mild-BPD *n* = 35 – Severe-BPD *n* = 15 – Excluded preterm infants *n* = 7 (Died in early days)	A prospective, single-centre study was conducted at a tertiary perinatal centre (2009–2014). Echo parameters were obtained *via* one of the following methods: – Pulsed wave (PW) Doppler. – Tissue Doppler Imaging (TDI). Appropriate statistical tests were used for analyses.
Czernik et al., 2012	Germany	Assessing the usefulness of the right ventricular index of myocardial performance (RIMP) to estimate pulmonary vascular resistance in very low birth weight infants.	A total of 143 preterm infants (<32 weeks gestation) were eligible for inclusion: Preterm infants included in the final analysis were *n* = 121: No-BPD *n* = 85 Preterm infants with BPD *n* = 36 Preterm infants were excluded from the final analysis were *n* = 22: Preterm infants with congenital heart disease *n* = 4. Preterm infants died within four weeks after birth *n* = 18.	A prospective, single-centre study was conducted at a tertiary perinatal centre from September 2008 till January 2010. The study was approved by the ethics committee at the institution. Echo parameters were obtained *via*: – Pulsed wave (PW) Doppler. Appropriate statistical tests were used for analyses.
Di Maria et al., 2015	USA	The aim was to prospectively study maturational changes in diastolic tissue velocities at two points in time; at seven days old and at 36 weeks post-menstrual age (PMA). Further analysis was performed to establish whether DTI measures were altered in infants with bronchopulmonary dysplasia (BPD) with or without pulmonary hypertension (PH).	Preterm infants were eligible for inclusion if they were ≤34 weeks gestation and had a birth weight between 500 and 1,250 grams. Enrolment is to take place within seven days after birth. The total number of enrolled preterm infants was 277 (274 received echo assessment at seven days after birth): Preterm infants included in the final analysis were: – Preterm infants without BPD *n* = 111. – Preterm infants with BPD *n* = 166.	A prospective, multi-centre trial was conducted from July 2006 till March 2012. The study team obtained ethical approvals from the participating institutions (University of Colorado Denver and Indiana University). Echo parameters were obtained *via* one of the following methods: – Pulsed wave (PW) Doppler. – Tissue Doppler Imaging (TDI). Appropriate statistical tests were used for analyses.
Helfer et al., 2014	Germany	The study aims to examine the development of cardiac function in preterm infants by measuring tissue Doppler-derived peak systolic strain (PSS) and strain rate (PSSR) in the first 28 days after birth. It is also to assess the impact of BPD on PSS & PSSR.	A total of 119 preterm infants (birth weight <1500 g) were included: Images from 110 preterm infants were suitable for analysis: – Preterm infants without BPD *n* = 79. – Preterm infants with BPD *n* = 31.	A prospective, single-centre trial was conducted at a tertiary perinatal centre from September 2008 to January 2010. The study was approved by the ethics committee at the research team institution. Echo parameters were obtained *via*: – Pulsed—Tissue Doppler Imaging (TDI). Appropriate statistical tests were used for analyses.
Levy et al., 2015	USA	The study aimed to determine the maturational (age- and weight-related) changes in the Right Ventricle fractional area of change (RV FAC) and RV areas and to establish reference values in healthy preterm and term neonates.	A total of 115 preterm infants (23–28 weeks of gestation) were enrolled: Preterm infants included in the final analysis were *n* = 115: – No or mild BPD *n* = 60 – Preterm infants with moderate-severe BPD *n* = 55.	A prospective, longitudinal and single centre was conducted at a tertiary perinatal centre from August 2011 till August 2013. The study was approved by the ethics committee at the institution. Echo parameters were obtained *via*: – 2D and RV-focused 4chambers (4ch) view. 2D speckle tracking. Appropriate statistical tests were used for analyses.
Levy et al, 2017	USA/Republic of Ireland	The study aims to determine the maturational changes in systolic strain mechanics of Ventricles by 2D speckle tracking echocardiography in extreme preterm neonates from birth to one year of age. Also, it assesses the impact of disorders, e.g., BPD, on the deformation measures.	A total of 239 preterm infants (23–28 weeks gestation) were enrolled; 17 participants died before discharge from the hospital, so only 222 preterm infants were included in the data analysis: Healthy preterm infants BPD *n* = 103. Preterm infants with the following diagnoses (*n* = 119): Preterm infants with BPD *n* = 116. Preterm infants with pulmonary hypertension *n* = 17. Preterm infants with PDA *n* = 100.	A prospective, longitudinal and multi-centre study was conducted at hospitals affiliated with two academic institutions (Washington University School of Medicine, Saint Louis Children's Hospital, and Royal College of Surgeons in Ireland, Rotunda Hospital) from August 2011 to January 2016. The study was approved by the institutional review board of Washington University, and the ethical committee on human research at the Royal College of Surgeons approved the protocol. Echo parameters were obtained *via*: – Two-Dimensional Speckle Tracking Imaging. Appropriate statistical tests were used for analyses.
Mendez-Abad et al., 2020	Spain	The study aims to assess the influence of BPD on the maturation of the myocardium in very low birth weight infants (VLBWIs).	Preterm infants born at ≤32 weeks gestation and/or ≤1500 g were eligible for inclusion. Preterm infants included in the final analysis were *n* = 101: – No-BPD *n* = 86 – Preterm infants with BPD *n* = 15	A prospective, observational, single-centre study was conducted at a tertiary perinatal centre from January 2015 to 2017. The study was approved by the ethics committee at the study institution. Echo parameters were obtained *via*: – Tissue Doppler Imaging (TDI). – M-Mode. Appropriate statistical tests were used for analyses.
Neumann et al., 2021	Germany/Switzerland	The study aims to examine the prognostic capability of early echocardiographic assessment of right ventricular function and vasoactive peptides to predict BPD or death in very preterm infants.	Preterm infants born at <32 weeks gestation were eligible for inclusion. Preterm infants included in the final analysis were *n* = 294: – No-BPD *n* = 229 – Preterm infants with BPD/death *n* = 65	A prospective, observational, multi-centre study was conducted at two tertiary perinatal centres (University Children's Hospital Basel, Switzerland and Charité Universitätsmedizin Berlin, Germany) from January 2015 to November 2017. The study was approved by the ethics committees at the two study institutions. Echo parameters were obtained *via* one of the following methods: – Pulsed wave (PW) Doppler. – M-Mode. Appropriate statistical tests were used for analyses.

**Table 3:** Selected studies details related to authorship, the timing, analysis and the key findings of the echo scans. .

Authors & Year	Timing of Echo Scans	Echo Parameters measured	Echo Parameters Calculation	Key Findings
Bokiniec et al., 2017	Echo scans were performed at three-time points: – First day after birth. – 28 days after birth. – 36 weeks of PMA.	– B was calculated from pulsed Doppler waveforms of TV inflow. – A was calculated from pulsed Doppler waveforms of RV outflow. – TV diastolic velocities; A & E waves. – TDI velocities, e.g.., (A’, E’, S’)	RV-MPI (Tei Index) was calculated *via*: RV-MPI (TDI) =IVCT + IVRT/ET RV-MPI (PW) =a−b/b. E/E’ RV ratio	Increased RV-MPI *via* PW-Doppler at 28 days after birth in preterm infants with severe-BPD group compared with both the preterm infants without BPD (*p *= .014) and the preterm infants with mild-BPD groups (*p *= .031). No difference in RV-MPI between preterm infants with no-BPD and preterm infants with mild-BPD groups was detected (*p *= .919). No difference in E/E’ RV ratio between the groups at any time point.
Czernik et al., 2012	Echo scans were performed at four-time points: – At the second day after birth. – At the 7th day after birth. – At two weeks after birth. – At the 28th day after birth.	– B was calculated from pulsed Doppler waveforms of TV inflow. – A was calculated from pulsed Doppler waveforms of RV outflow.	RV-MPI (Tei Index) was calculated *via*: RV-MPI = a−b/b.	– RV-MPI was decreased in the last three echo scans (7th, 14th & 28th days old) in preterm infants without BPD, while remained elevated in those infants who are affected by BPD; 7 days old: 0.31[0.22–0.39] vs. 0.35[0.29–0.48], *p *= 0.014; the 14 days old: 0.23[0.17–0.30] vs. 0.35[0.25–0.43], *p* = 0.001 and 28 days old: 0.21[0.15–0.28] vs. 0.31 [0.21–0.35], *p* = 0.015).
Di Maria et al., 2015	Echo scans were performed at two-time points: – At the 7th day after birth. (*n* = 274) – At 36 weeks PMA. (*n* = 277)	– RV-free Wall—E’ (cm/sec) – RV-Free Wall-A’ (cm/sec) TV diastolic velocities; A & E waves.	– RV Free Wall-E’/A’ – TV E/E’	When grouping the study participants by BPD or PH status, there was no significant association between TDI measures and the presence or absence of PH or BPD.
Helfer et al., 2014	Echo scans were performed at four-time points: – At the first day after birth (0–3). – At the 7th day after birth (6–10). – At two weeks after birth (12–17). – At the 28th day after birth (22–31).	– Peak Systolic Strain (PSS) of RV-free walls. – Peak Systolic Strain Rate (PSSR) of RV-free walls.	N/A	In preterm infants who developed BPD, PSS was significantly lower on days 14 and 28.
Levy et al., 2015	All the study participants (*n* = 115) had echo scans performed at two-time points; 32 and 36 weeks of PMA. *Thirty (30)* of the participants had additional echo scans at two-time points, 24 and 72 h after birth, i.e., these infants got *four echo scans* in total.	Right Ventricle fractional area of change (RV FAC).	RV-FAC = 100 × [RV end-diastolic area (cm^2^)—RV end-systolic area (cm^2^)]/ RV end-diastolic area (cm^2^).	From one to three days of age, there was no difference in right ventricle end-diastolic area, RVEDA and right ventricle end-systolic area, or RVESA between the reference cohort (infants with mild or no BPD) and the infants with moderate or severe BPD. By 32 weeks of PMA, there was a statistically significant increase in RVEDA and RVESA (*p* = 0.034) amongst infants with moderate or severe BPD.
Levy et al., 2017	All the study participants had echo scans performed at four-time points, in addition to one follow-up echo at one (1) year of corrected age: 1, 2, 5–7 days after birth and at 32 and 36 weeks of PMA. At the Washington University School of Medicine site, echo scans were performed at: Day 1, *n* = 30. Day 2, *n* = 30. 32 weeks PMA, *n* = 117. 36 weeks PMA, *n* = 117. At the Royal College of Surgeons in Ireland site, echo scans were performed at: Day 1, *n* = 102. Day 2, *n* = 102. Day 5–7, *n* = 98. 36 weeks PMA, *n* = 47.	RV FWLS (%) = RV—Free Wall Longitudinal Strain. RV FWLSRs (1/s) = RV—Free Wall Longitudinal Strain Rate. RV SLS (%) = RV—Segmental longitudinal strain. IVS GLS (%) = Intraventricular Septum (IVS) Global Longitudinal Strain. IVS GLSR (1/s) = IVS Global Longitudinal Strain Rate. IVS SLS (%) = IVS Segmental longitudinal strain.	N/A	IVS SLS with preterm infants with BPD and/or PH remains in an apex-to-base pattern (highest to lower), reflective of RV regional gradient. Preterm infants who developed BPD not only had decreased RV- GLS and IVS GLS from 32 weeks PMA to one year CA, but a persistent base-to-apex (reflective of an RV dominant pattern) IVS strain gradient that never reversed, even by one year of age.
Mendez-Abad et al., 2020	Echo scans were performed at numerous time points; 24 and 72 h old, then weekly until 36 weeks of PMA. N-terminal pro-B type natriuretic peptide (NTproBNP) plasma level on the 14th day after birth.	Early diastolic (E′) velocity. Late diastolic (A′) velocity. Tricuspid Annular Plane Systolic Excursion (TAPSE).	N/A	There is a negative correlation between the plasma NTproBNP levels and TAPSE (Spearman's Rho = −0.36, *p* = 0.0001), with similar results regarding diastolic velocities derived of TDI. Myocardial function maturation in VLWBIs that develop BPD seems to be delayed.
Neumann et al., 2021	Echo scan and blood tests were performed on the 7th day after birth. Blood tests to measure the Plasma concentrations of mid-regional pro-atrial natriuretic peptide (MR-proANP) and C-terminal pro-endothelin-1 (CT-proET1), were performed at the same time of the echo scans.	– TAPSE. – B was calculated from pulsed Doppler waveforms of TV inflow. – A was calculated from pulsed Doppler waveforms of RV outflow.	RV-MPI (Tei Index) was calculated *via*: RV-MPI = a−b/b.	RV-MPI values were higher, and TAPSE values were lower in infants with BPD/death than in infants without BPD. Both vasoactive peptides (MR-proANP and CT-proET1) were significantly higher in BPD/death infants as compared to controls.

### 3.2. Narrative description of studies

The eight included studies were conducted in the USA and European countries. All studies were conducted in tertiary perinatal centres. Only four studies examined exclusively preterm infants born before 32 weeks of gestation (11, 13–15) (Table 1). The study by Helfer et al., 2014, included preterm infants born at 32 weeks gestation as the recruitment was per birth weight rather than gestational age (16). Another study included preterm infants with a higher gestation age, and the birth weight needed to be within a specific range (≤34 weeks gestation and birth weight between 500 and 1,250 grams) (17). The other two studies by Levy and colleagues focused on recruiting preterm infants with lower gestational age (23–28 weeks of gestation) (18, 19).

Different definitions to diagnose BPD were used in the selected eight studies. Czernik et al., 2012, diagnosed preterm infants with BPD based on their oxygen requirement to maintain pre-ductal arterial saturations of 92% at 36 weeks PMA (20, 21). Levy et al., 2017, utilized a modified Shennan definition (22). While the remaining six studies used the definition set in the NIH workshop (23). There is a lack of the definition of an early BPD, i.e., during the first two weeks after birth, in the selected studies.

#### 3.2.1. What are the techniques used to capture the echo images

Several techniques were used to capture the echo images in the selected studies, such as 2D echo images, Pulsed wave (PW) Doppler, Tissue Doppler Imaging (TDI), 2D speckle tracking and M-mode.

#### 3.2.2. When were the echo scans performed in the first two weeks after birth?

The studies' timing and frequency of echo scans varied (Table 2). Three studies performed one echo scan within the first two weeks after birth (13, 15, 17); one study had the echo scans performed on the first day after birth (DAB) (17), and the other two studies performed the echo scans at the 7th DAB, respectively (13, 15). The remaining five studies had several echo scans performed within the first two weeks after birth (11, 14, 16, 18, 19). There were variations between the studies regarding the timing when the echo scans were performed in the first 14 days after birth; Czernik et al., 2012 trial, the echo scans were performed on day 2, 7, 14 after birth (11); Helfer et al., 2014 study team performed the echo scans on day 1, 7, 14 after birth (16); Levy et al., 2015 study team performed the echo scans on day 1 and day 7 after birth (18); Levy et al., 2017 study teams performed the echo scans on day 1, 2, 5 and 7 after Birth (19); finally, Mendez-Abad et al., 2020 team per, the echo scans were performed on day 1, 3, 7, 14 after birth (14).

#### 3.2.3. What echo parameters are used to assess the haemodynamic effects of early BPD on the function of the right ventricle?

The critically appraised studies utilised different echo parameters to assess the function of the RV in healthy preterm infants (controls) and the ones affected by BPD (cases); RV-myocardial performance index (MPI), RV strain (S) and strain rate (SR), RV fractional area of change (RV-FAC), TDI systolic- and diastolic-velocities and tricuspid annular plane systolic excursion (TAPSE).

#### 3.2.4. How were the echo parameters measured and analysed?

Studies utilising RV-MPI to assess RV function, the calculation of RV-MPI was performed by using one of the following two equations:

– RV-MPI calculation in TDI echo images: RV-MPI (TDI) = IVCT + IVRT/ET.
  (IVCT = Isovolumic Contraction Time; IVRT = Isovolumic Relaxation Time; ET = Ejection Time)
– RV-MPI calculation in PW echo images: RV-MPI (PW) = a−b/b.
  (a = the measurement between cessation and onset of the tricuspid valve inflow; b = the ejection time of the RV outflow)

Four studies showed a delay in the normal maturation of the RV-MPI, i.e., persistently raised RV-MPI in BPD-affected preterm infants in comparison to the controls. The differences in the RV-MPI between the two groups (preterm infants with and without early BPD) were detected in the echo scans performed after the first three days after birth (11, 13–15). In addition, Neumann et al., 2021, demonstrated that TAPSE is lower in BPD-affected preterm infants (15).

Other findings were identified when examining all the included studies. Neumann et al., 2021, demonstrated that TAPSE is lower in BPD-affected preterm infants (15). Levy et al., 2015, showed differences in the RV-FAC measurements between controls and BPD-affected preterm infants when assessed at 32 weeks of PMA echo scans (18). Two studies utilising S and SR also demonstrated statistically significant differences in these measurements between their controls and the cases of preterm infants (16, 19). However, the study by Helfer et al., 2014, demonstrated that the differences in the S & SR measurements between controls and BPD-affected preterm infants would start to manifest in the echo scans at day 14 after birth while the study of Levy et al., 2017, the S & SR differences between the controls and the cases of preterm infants with BPD, did not manifest till the echo scan at 32 weeks of PMA (16, 19).

In addition to using echo parameters, two of the eight studies performed blood tests. Mendez-Abad et al., 2020, analysed N-terminal pro-B type natriuretic peptide (NTproBNP) plasma level on day 14 after birth (14). Neumann et al., 2021, analysed the plasma concentrations of mid-regional pro-atrial natriuretic peptide (MR-proANP) and C-terminal pro-endothelin-1 (CT-proET1) on day 7 after birth (15). Both studies demonstrated a statistically significant difference in the plasma levels of the selected tests between the controls and BPD-affected preterm infants.

## 4. Discussion

There were methodological variations between the selected studies. There are differences in the number and the timing of echo scans performed in the first two weeks of postnatal life, together with the variations in the echo parameters used to compare the preterm infants as controls and the preterm infants affected by BPD.

Echo scans performed in the first three days after birth did not demonstrate a difference in the echo parameters measurements between the control group and preterm infants with early BPD. In comparison, significant differences in the echo parameters measurements between the controls and preterm infants with BPD were seen in the echo scans performed at the end of the first week after birth and the subsequent weeks (11, 13, 15, 16). Similarly, the study by Levy et al., 2015, showed that RV-FAC measurements in the first three days after birth did not differ between preterm infants without BPD and the ones who have BPD (18).

Studies using RV-MPI to identify RV dysfunction associated with early BPD have similar findings. Bokiniec et al., 2017, showed that PW-Doppler could identify the differences in RV-MPI between controls and preterm infants affected by early BPD, while TDI could not demonstrate differences in RV-MPI between preterm infants with and without BPD (13). The trials conducted by Czernik et al., 2012, and Neumann et al., 2021 showed similar results when RV-MPI *via* PW-Doppler could identify RV dysfunction in preterm infants with early BPD (11, 15). While Mendez et al. 2020 findings demonstrated that RV-MPI and diastolic velocities *via* TDI can be still a useful tool since it could detect the cardiac maturational changes in preterm infants. The same study showed that diastolic velocities *via* TDI are capable of recognising RV dysfunction in infants with early BPD (14).

Despite the limitations of PW-Doppler in calculating RV-MPI, e.g., it utilises the measurements from different cardiac cycles, three of the eight selected studies used PW-Doppler and were able to diagnose RV dysfunction in preterm infants with early BPD. On the other hand, despite TDI being a valuable and practical tool in assessing RV's systolic and diastolic function, TDI was unable to identify the RV dysfunction (13, 17). This could be explained by the inherent limitations of TDI, which is angle-dependent and influenced by the global cardiac motion and the inability to differentiate between passive and active motion (24). In addition, velocities and displacements depend on the cardiac sizes; hence, larger hearts have higher velocities and displacements. Therefore, it is crucial to normalise the TDI measurements according to the cardiac size (25). None of the studies that utilised TDI technique normalised the cardiac TDI measurements, which might negatively affect the ability of these studies to demonstrate a significant difference in the TDI measurements between controls and cases.

Speckle tracking can be used to measure S and SR and diagnose RV dysfunction. However, the findings of the studies that utilised speckle tracking in this review varied. A study by Levy et al., 2017, could not diagnose the RV dysfunction in the echo scans performed at day 1, 2, and 5 to 7 after birth (19). In contrast, a study by Helfer et al., 2014, demonstrated a significant difference in the S and SR between preterm infants with and without BPD when echo scans were performed at day 14 after birth (16). The findings of these two studies demonstrate that results can vary even when a reliable technique such as speckle tracking was used. This might be well related to the timing of the echo scans, i.e., if the echo scan were performed too soon, it might not capture the hemodynamic changes related to RV dysfunction that is associated with early BPD.

Furthermore, two of the selected eight studies added blood tests to their diagnostic model of early BPD (14, 15). Both studies demonstrated that adding specific blood tests would enhance the diagnostic ability of RV dysfunction associated with early BPD (14, 15).

Although, adding a specific blood test to a diagnostic model might enhance its diagnostic ability, it might also have a negative impact on its applicability in many perinatal centres worldwide, especially in developing countries, due to, for example, the cost and unavailability of these blood tests. Likewise, it will negate the idea of having a diagnostic model based on a practical, applicable and non-invasive test such as an echo scan.

The primary strength of this review was its broad literature search. The process of synthesising the scientific evidence was transparent and systematic in identifying and mapping the evidence related the use of echocardiography to diagnose RV dysfunction associated with early BPD. Nevertheless, as this review designed as a systematic scoping review, it did not assess the quality of the evidence, so it does not provide the scientific basis to inform clinical decision making. Finally, language restrictions may have led to the exclusion of a few studies.

## 5. Conclusion

This scoping review showed the need to develop a diagnostic model based on a non-invasive echo scan. The review demonstrated the emergence of unanswered questions regarding the practicalities of when and how many echo scans are needed and which cardiac parameter is required to diagnose early BPD in preterm infants.

BPD will adversely affect the myocardium function of the RV; these negative influences can be captured in the first two weeks of postnatal life (15, 17). However, there are still knowledge gaps regarding the appropriate number, timing of the echo scans and the most suitable cardiac parameters to assess RV function.
